# Blow me down: A new perspective on *Aloe dichotoma* mortality from windthrow

**DOI:** 10.1186/1472-6785-14-7

**Published:** 2014-03-18

**Authors:** Samuel Linton Jack, Michael Timm Hoffman, Rick Frederick Rohde, Ian Durbach, Margaret Archibald

**Affiliations:** 1 Plant Conservation Unit, Department of Biological Sciences, University of Cape Town, Cape Town, South Africa; 2 Centre of African Studies, University of Edinburgh, Edinburgh, UK; 3 Department of Statistical Sciences, University of Cape Town, Cape Town, South Africa; 4 Department of Mathematics and Applied Mathematics, University of Cape Town, Cape Town, South Africa

**Keywords:** Aloe dichotoma, Arid environment, Convective rainfall, Indicator species, Mortality, Southern Africa, Wind speed, Windthrow

## Abstract

**Background:**

Windthrow, the uprooting of trees during storms associated with strong winds, is a well-established cause of mortality in temperate regions of the world, often with large ecological consequences. However, this phenomenon has received little attention within arid regions and is not well documented in southern Africa. Slow rates of post-disturbance recovery and projected increases in extreme weather events in arid areas mean that windthrow could be more common and have bigger impacts on these ecosystems in the future. This is of concern due to slow rates of post-disturbance recovery in arid systems and projected increases in extreme weather events in these areas. This study investigated the spatial pattern, magnitude and likely causes of windthrown mortality in relation to other forms of mortality in *Aloe dichotoma*, an iconic arid-adapted arborescent succulent and southern Africa climate change indicator species.

**Results:**

We found that windthrown mortality was greatest within the equatorward summer rainfall zone (SRZ) of its distribution (mean = 31%, n = 11), and was derived almost exclusively from the larger adult age class. A logistic modelling exercise indicated that windthrown mortality was strongly associated with greater amounts of warm season (summer) rainfall in the SRZ, higher wind speeds, and leptosols. A statistically significant interaction term between higher summer rainfall and wind speeds further increased the odds of being windthrown. While these results would benefit from improvements in the resolution of wind and substrate data, they do support the hypothesised mechanism for windthrow in *A. dichotoma*. This involves powerful storm gusts associated with either the current or subsequent rainfall event, heavy convective rainfall, and an associated increase in soil malleability. Shallow rooting depths in gravel-rich soils and an inflexible, top-heavy canopy structure make individuals especially prone to windthrown mortality during storms.

**Conclusions:**

Results highlight the importance of this previously unrecognised form of mortality in *A. dichotoma*, especially since it seems to disproportionately affect reproductively mature adult individuals in an infrequently recruiting species. Smaller, more geographically isolated and adult dominated populations in the summer rainfall zone are likely to be more vulnerable to localised extinction due to windthrow events.

## Background

Windthrow, which refers to trees being broken or uprooted by the wind, is a well established cause of catastrophic mortality in plantations and natural forests, often with large economic and ecological consequences [1,2]. Analyses of the impacts of windthrown mortality have typically been restricted to tropical forests and northern hemisphere temperate forests e.g. [3-7]. Very few studies have documented the ecological effects of windthrown mortality on species in more open, arid environments (but see [8] for a savanna example with a social focus).

Understanding the effects of windthrow in arid ecosystems is important for two reasons. Firstly, plant community recovery from disturbance, such as windthrown mortality, is typically more prolonged in arid systems due to resource limitations such as a lack of moisture and, therefore, access to nutrients. Resource limitations reduce growth rates and extend the intervals during which phases of reproduction and establishment are likely to occur [9,10]. Added anthropogenic pressure in arid regions come in the form of landscape fragmentation, heavy grazing pressure, and water abstraction, which combine to further retard rates of recovery [11].

Secondly, global climate change models predict an increase in the intensity and a decrease in the return interval of extreme weather events [12]. Notwithstanding evidence for a counter-intuitive decline in global *average* windrun [13], an increased intensity of storm events is likely to be accompanied by increased precipitation and localised wind speeds in certain areas e.g. [14], with implications for windthrown mortality, as has been postulated for European forest stands [15,16].

The current study investigated the role of windthrown mortality in *Aloe dichotoma*, a widespread and iconic succulent tree species distributed in the arid and semi-arid winter and summer rainfall regions of Namibia and South Africa. Previous research has emphasized the role that increasing temperature, drought, and biotic factors such as herbivory and disease have played on the high levels of mortality observed in many populations of this species [17-19]. Despite observational evidence that windthrow contributes a large fraction to total mortality in some *A. dichotoma* populations, this phenomenon has not been investigated. Indeed, an explicit analysis of windthrown mortality does not exist for any southern African species, despite the fact that conditions likely to promote windthrow events (i.e. convective storm events with associated high wind speeds) are ubiquitous in the summer rainfall zone (SRZ) which dominates southern Africa e.g. [20]. In addition, southern Africa appears to be an area where extreme weather events are predicted to increase in frequency [21,22].

Studies in both temperate and tropical settings suggest that taller, larger individuals with greater canopy surface areas are likely to be more susceptible to windthrown mortality [23,24]. This is due chiefly to greater drag and reduced stem flexibility associated with large individuals [25,26], although other architectural factors such as reduced canopy density, increased branch flexibility, and a more aerodynamic leaf-shape can have a mitigatory effect [27]. This may be cause for concern when considering *A. dichotoma*, as the species has a characteristically rigid stem, canopy branch and leaf architecture. In addition, smaller and more climatically marginal populations, which are often dominated by large, mature adult individuals, may be especially at risk. Adult individuals typically bear a disproportionate amount of the reproductive potential in a population and windthrow events which increase adult mortality can therefore have a detrimental effect on long term population persistence, especially in areas where recruitment is infrequent.

Soil water content, as well as rooting structure, depth, and substrate, are important additional determinants of windthrow susceptibility. An increase in soil water content, for example, reduces soil resistance to slippage [28], while an increase in the adhesive ability of soil particles to water presumably prolongs the period of vulnerability to windthrow [29]. Generally, thicker root plates (i.e. the vertical rooting extent) which tap deeper water sources in well drained soils provide a greater resistive force against shearing [30]. The less horizontally extensive and shallower root plates typical of adventitious root systems associated with monocotyledonous plants such as *A. dichotoma* may, therefore, be more susceptible to shearing at the root-soil interface, particularly if the soil at the base of the root plate (and below this level) is saturated. This phenomenon has been well demonstrated for the root anchorage of *Chamaecyparis obtusa* (Hinoki), a cypress species native to central Japan [28].

Given the general lack of knowledge pertaining to this phenomenon in semi-arid southern African, the current study provides baseline information on the spatial pattern and demographic characteristics of windthrown mortality in *A. dichotoma*. It also establishes the percentage of mortality resulting from windthrow, rather than from other possible causes, such as physiological and climatological drought e.g. [17,19,31]. Lastly, we relate the pattern of windthrown mortality to potential causative factors such as tree size, rainfall characteristics, wind speed, geology and lithology. In doing so, we identify areas where populations might be more vulnerable to windthrow, and therefore require more active management to ensure their continued survival.

## Methods

### Data collection and classification

Fourteen *A. dichotoma* populations were sampled over two consecutive winter seasons in Namibia and the Northern Cape in South Africa during 2008 and 2009 (Table 1). As far as possible, populations were chosen at roughly equal intervals spanning the latitudinal extent and thus the climatic range of the species’ distribution. Populations with individuals on all four aspects were also selected so as to capture the full range in local conditions due to micro-topographical effects. Where possible, approximately 240 (60 per aspect) individuals (both living and dead) were recorded along altitudinal transects within a population. Conspicuous populations along busy transport routes easily accessed from the roadside were avoided due to the likelihood of prior removal of juveniles or (potentially windthrown) dead skeletons for firewood or building purposes.

**Table 1:** **Numbers of sampled *Aloe dichotoma *** **individuals per population and basic climate characteristics**

**Population name**	**Total number of individuals counted**	**Ave. altitude (m) (±stdev)**	**Ave. rainfall (mm) (±stdev)**	**Ave. temperature (°C) (±stdev)**
Brandberg	177	1666 (±277)	204.3 (±41.8)	17.3 (±1.4)
Omaruru River	185	734 (±25)	100.8 (±4.2)	21.8 (±0.1)
Spitzkoppe	162	1231 (±86)	135.1 (±9.9)	20.1 (±0.3)
Tinkas River	258	699 (±25)	82.2 (±2.4)	20.6 (±0)
Remhoogte	211	1411 (±89)	189.4 (±9.4)	16.9 (±0.5)
Hauchabfontein	290	1226 (±55)	124.3 (±0.5)	16.6 (±0)
Gorab	274	1470 (±48)	138.6 (±9.5)	15.5 (±0.3)
Namtib	293	1265 (±59)	102.8 (±2.9)	15.4 (±0.1)
Carolinahof	222	1273 (±12)	134 (±1.5)	17.2 (±0.1)
Kliphoek	293	1262 (±36)	101.5 (±4.4)	15.5 (±0.2)
Grunau	241	913 (±17)	103 (±0)	19.2 (±0)
Bulletrap	245	846 (±30)	143.9 (±3.9)	17.6 (±0.1)
Rooifontein	243	788 (±9)	188.9 (±4.7)	17.2 (±0.2)
Gannabos	274	461 (±23)	204.7 (±8.9)	18.3 (±0.2)

Numbers of *Aloe dichotoma* individuals counted per population and climate characteristics derived for each of the 14 sampled populations in Namibia and South Africa. Populations above and below the bold line fall in the summer and winter rainfall zones, respectively.

GPS co-ordinates and altitude were recorded for every individual and these were used to extract average climatic conditions at each population from the well established and highly resolved (±1 km) interpolated global climate dataset, Worldclim (Table 1) [32]. A number of allometric measurements were taken for each individual, including basal circumference, circumference at first branching node, total height and canopy diameter. Trees were then subjectively assigned to juvenile, young adult, mature adult, senescent or dead age class categories based on allometric criteria outlined in Table 2. These five categories were later reduced to three for ease of analysis and interpretability.

**Table 2:** **Age class categories for *Aloe dichotoma *** **individuals based on architectural and reproductive characteristics**

**Age class**	**Original age class**	**Plant height (m)**	**Canopy diameter (m)**	**Dichotomous branching nodes**	**Leaf rosettes**	**Reprod. mature**
Juvenile	Juvenile	Generally <1.5	ca. 0.60	None	1	N
	Young adult	Variable, generally 2-3	1-1.5	1-3	2-8	Y
Adult	Mature adult	Variable, generally 3–5.5	2-3.5	6-10	>10	Y
Dead	Senescent	Generally >4	2-4	8-12	>20	Y
	Dead	n/a	n/a	n/a	n/a	n/a

**Figure 1:**
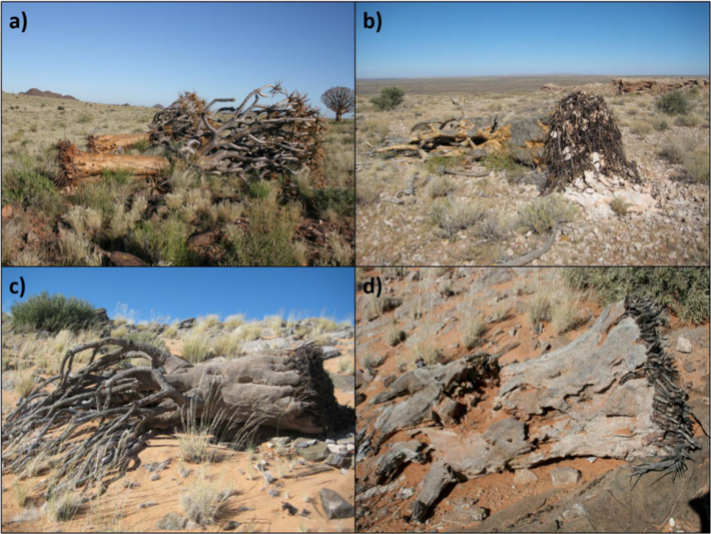
**Examples of windthrown individuals of increasing ‘time since death’, read from most recent (a) to the oldest (d).** Note the parallel orientation of the recently fallen trees in **(a)**, the leveraged depression where the tree was previously rooted and **(b)**, the connection of branches to the main stem in **(a)** to **(c)**, and the exposed roots attached to the base of the stem in all the images.

Dead individuals were grouped into windthrown and non-windthrown categories based on two main lines of evidence. Firstly, windthrown individuals were always found lying on their sides, with attached canopies in all but the oldest dead skeletons (Figure 1). In addition, no alternative explanation could be found for why an individual had fallen over, such as stem or branch damage which might suggest an animal had pushed it over, or being situated on a very steep and unstable slope where toppling over could occur without assistance from the wind. Multiple fallen trees orientated in a similar direction was not considered an essential criterion for being designated as windthrown. This was because an often complex topography was likely to cause winds associated with discrete storm events to gust in different directions, which over time would result in an accumulation of different fall directions, and windthrown trees on even gentle to moderately steep slopes sometimes rolled downslope, thereby altering their final orientation.

Secondly, windthrown individuals invariably had roots attached to the base of the trunk, irrespective of the ‘time since death’. In more recent windthrown examples this was often accompanied by soil and rocks still bound in the exposed root mass, as well as a depression created through the upward leverage of this material. If a tree lying on its side had decayed to the extent that the attached roots were no longer visible, it was excluded from the counts.

Although the possibility exists that individuals could be blown over *after* death, and therefore be incorrectly classified, this is thought to be a rare occurrence for two reasons. Firstly, upon death, individuals soon lose water stored in their stems, branches and leaves, making them less top-heavy and therefore prone to toppling over as a result of prior listing or wind gusts. In addition, the desiccation of rooting material after death seems not to diminish overall rooting strength to any great extent. Where individuals *have* toppled over for whatever reason, there is an additional way of determining – through inspection of the location of the root plate - if the toppling occurred before or after death. This is because the root plate is extremely well attached to the stem-base in living individuals, but the strength of this bond diminishes subsequent to mortality. Therefore, a root plate still attached to a long dead fallen tree is usually indicative of prior windthrown mortality, while an in situ root plate, separated from the stem-base, invariably means that toppling occurred after death.

A second reason to question the prevalence of post-death windthrow is that leaves and terminal branches are lost soon after death, which significantly reduces the drag force in the rigid canopy. Dead individuals were placed in the non-windthrown category if the dead stems were upright and rooted in the ground. The process of non-windthrown mortality (i.e. the shift from senescence to death) is illustrated in [19].

### ‘Time since death’

Estimates of the ‘time since death’ of both windthrown and non-windthrown or ‘standing’ dead individuals were based on long-term decay rates observed in several repeat photographs (M.T. Hoffman, unpublished observations), as well as on assessments of decay based on repeated visits to individuals known to have died recently. Six categories were used to classify ‘time since death’, using measures of structural integrity outlined in Table 3.

**Table 3:** Criteria used for estimating the number of years since individuals died

**Category**	**Years since death**	**Leaves**	**% bark remaining**	**Branches**	**Stem decay**	**Growth rings**	**% plant remaining**
1	1-5	Present	>95%	All; intact	None	Not visible	100%
2	6-10	Sometimes; dried out	50-75%	All; seldom intact	None	Not visible	90-95%
3	11-20	Absent	<40%	All; not intact	Slight	Not/barely visible	80-90%
4	21-40	Absent	<5%	>50% lower only	<25%	Becoming visible	~50%, usually trunk
5	41-60	Absent	Absent	<10% lowest only	25-50% or more	Clearly visible	~20-40%, trunk only
6	61+	Absent	Absent	Absent	>80%	Clearly visible	<5%, trunk only

### Tree surface area

**Figure 2:**
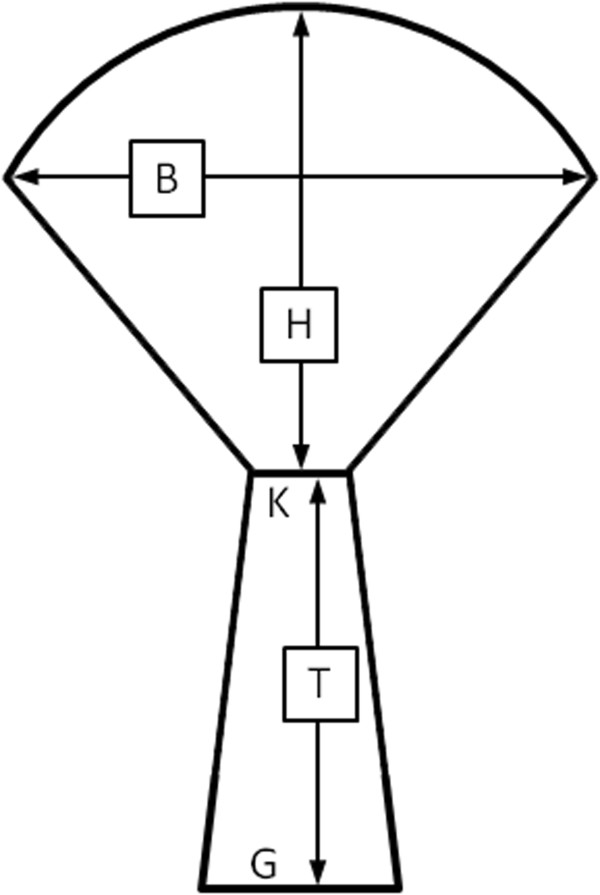
**Simplified shape of *Aloe dichotoma *** **for determining surface area, with dimensions outlined in the text.**

Individual tree surface area was calculated using allometric measurements and by representing the tree trunk and canopy as simplified geometric shapes. The trunk was simplified to a trapezium and the canopy to the sector of a circle with the triangular base removed (Figure 2). The equations used to derive surface areas are presented below. Average surface area based on all living adult individuals in a population was then graphed to determine whether a relationship existed between larger average surface area in a population and the percentage of windthrown mortality.

Area of the trunk = (K + G)T/2 and,

$$\frac{\begin{array}{l} \mathrm{Area}\;\mathrm{of}\;\mathrm{the}\;\mathrm{canopy}=\\ K^{2}\;\left(K^{2}-B^{2}\right)\;\left(2\mathrm{HK}+S\right)+2B^{2}\;\left(B^{2}\left(4H^{2}-K^{2}\right)+K\;\left(4H^{2}K+K^{3}+4\mathrm{HS}\right)\;\mathrm{ArcTan}\right)\;\left[\frac{\left(B-K\right)\left(B+K\right)}{2\mathrm{HK}+S}\right] \end{array}}{8{\left(B-K\right)}^{2}{\left(B+K\right)}^{2}}$$

where, $S\sqrt{B^{2}\left(K^{2}-B^{2}+4H^{2}\right)},$ K is the diameter at first branching node, G is the basal diameter, T is the height to first branching node, B is the canopy diameter, and H is canopy height.

### Rainfall, wind-speed and lithology

A range of climate variables extracted from the interpolated Worldclim dataset [32] were individually regressed against the percentage of windthrown mortality in a population. Variables included mean precipitation of the warmest, coldest, wettest and driest months and quarters, as well as precipitation variability. Several measures of wind speed were derived from daily wind data for Namibia for the longest time period possible, and only included stations positioned as close to sampled *A. dichotoma* populations as possible. The most detailed geological and lithological maps available were used to describe the rooting substrate at each population.

### Modelling procedure

A logistic regression model with the following form was fitted to the available environmental data:

$$\log\;\left(\frac{p}{1-p}\right)=\beta_{0}+{\sum_{i}^{I}\beta_{i}c_{i}+\sum_{j=1}^{J}\gamma }_{j}d_{j}+\in$$

where p is the probability of death by windthrow, c_i_ are independent climatic variables, d_j_ are independent ‘demographic’ variables (indicators for geology and lithology, and basal circumference), and ∈ is an error term.

To facilitate the interpretation of main and interaction effects, all of the continuous independent variables were standardized to have zero mean and unit standard deviation [33]. A stepwise approach was used to find significant main effects, following which all second-order interactions between significant main effects were tested for significance. Only one of the interactions (between mean precipitation of the warmest quarter (PwarmQ) and the average of the 5 windiest days per month for the windiest time of day) was significant at a 5% level and hence included in the model.

## Results

### Percentage of windthrown mortality

**Figure 3:**
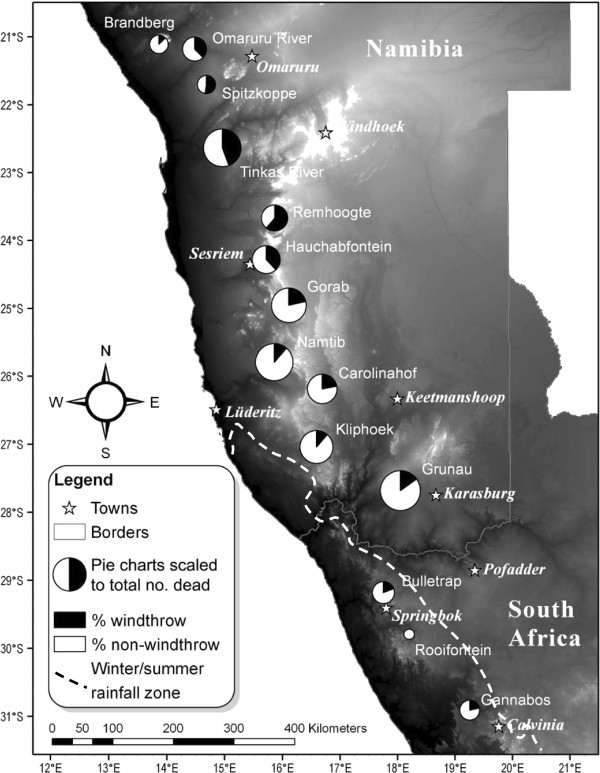
**Percentage of windthrown to non-windthrown deaths for each of 14 *Aloe dichotoma *** **populations.**

**Figure 4:**
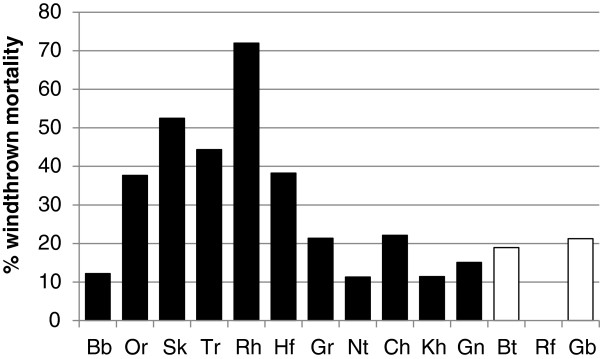
**Percentage windthrown A*loe dichotoma *mortality in 14 populations, arranged from equatorward to poleward range extent.** Black and white bars fall in the summer and winter rainfall zones, respectively. (See Table 1 and Figure 1 for the full names of the sampled populations).

Windthrow was a commonly observed form of mortality throughout the latitudinal distribution of *A. dichotoma* (Figure 3), but especially in populations occurring in the middle to northern reaches of the summer rainfall zone (SRZ) between the Omaruru River and the farm Hauchabfontein (Figure 4).

Average windthrown mortality across the entire SRZ accounted for 31% (n = 11) of total mortality, peaking at as much as 72% at Remhoogte. Windthrow incidence within the SRZ was much lower at the high elevation Brandberg population and south of Hauchabfontein, remaining low within the winter rainfall zone (WRZ). Here it accounted for only 13% of total mortality, although this figure was derived from only three populations. There were no windthrown individuals at Rooifontein (Figure 4), a population which also registered the lowest number of dead trees at thirteen (Figure 3).

### Windthrow and age class

**Figure 5:**
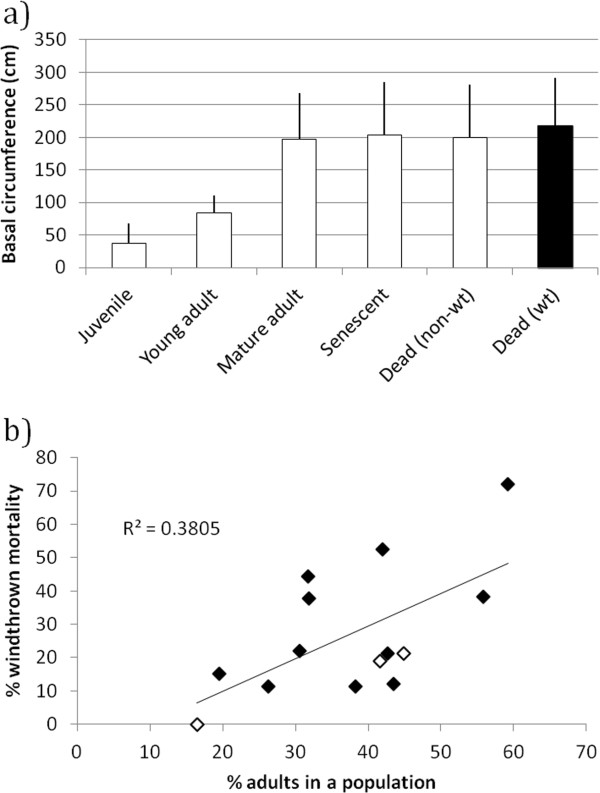
**(a) An illustration of basal circumference differences between non-windthrown living and dead individuals and windthrown dead and (b) the relationship between the percentage of adults in a population and windthrown mortality. (a)** Bars represent average basal circumferences for age classes with standard deviations attached. White bars represent non-windthrown living and dead individuals, and black bars windthrown dead individuals. **(b)** Black and white diamonds represent summer and winter rainfall zones, respectively.

All age classes had significantly smaller basal circumference measurements compared to the windthrown dead category (Figure 5a). However, the mean (M) and 95% confidence interval (CI) for the mature adult age class was comparable to that of windthrown dead (M = 197.2, CI = [193.4;201.0] and M = 218.2, CI = [210.7;225.7]), while it was an order of magnitude smaller for the combined juvenile and young adult age class (M = 50.8, CI = [47.9;53.6]). In addition, a positive relationship was found between the percentage of adult individuals in a population and the percentage of windthrown individuals (Figure 5b), indicating that windthrown mortality is more likely to be derived from the larger adult age class.

### ‘Time since death’

**Figure 6:**
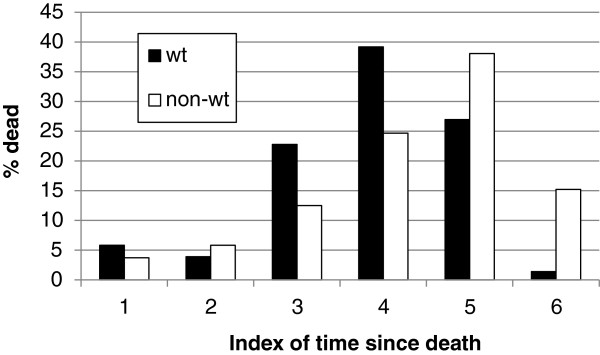
**The percentage of windthrown and non-windthrown mortality, assigned to an index of ‘time since death’.** Refer to Table 3 for a detailed description of structural criteria used in establishing ‘time since death’ categories.

Categorisation of the approximate ‘time since death’ suggested that windthrown individuals died slightly more recently than non-windthrown or ‘standing’ dead, although both categories indicated that mortality peaked some time ago, between 21–40 and 41–60 years before present (Figure 6).

### Possible drivers of windthrown mortality

**Figure 7:**
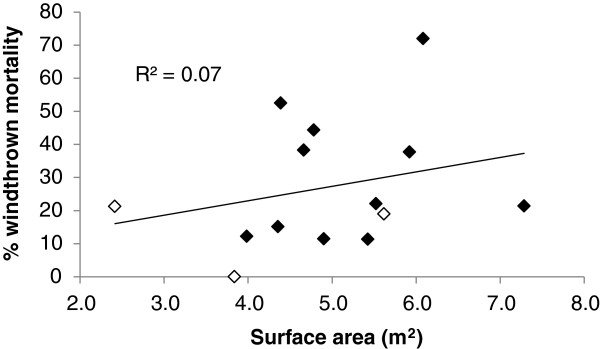
**Relationship between average tree surface area of mature adult and senescent individuals and percentage of windthrown individuals per population.** Black and white diamonds represent summer and winter rainfall zones, respectively.

Despite several prior studies linking greater surface area to increased windthrow susceptibility, the relationship between average tree surface area and percentage of windthrown individuals in the 14 sampled *A. dichotoma* populations was poor, with an R^2^ value of only 0.07 (Figure 7).

Wind speeds differed considerably between recording stations, especially between coastal and inland stations, with Diaz Point (a coastal station) registering the highest average and maximum wind speed (Table 4). Conversely, the inland station of Mariental had the lowest average and maximum wind speed. It was decided to use values for the average of the 5 windiest days per month in subsequent analyses as this was a compromise between annual average, which would not highlight extreme events, and maximum wind speed, which might not always coincide with rainfall events. However, interpretation of wind speeds in the context of windthrown death should be made with caution due to short recording periods in some cases (e.g. Omaruru), as well as the poor spatial network of stations collecting wind speed data and consequently, the large distances between stations and *A. dichotoma* populations.

**Table 4:** **Namibian wind speed records from stations nearest to *Aloe dichotoma *** **populations**

**Population**	**Nearest station**	**Dist. to nearest popn (km)**	**No. years on record**	**Quarter used to derive averages**	**Ave. ann. wind speed (m/s)**	**Ave. of the 5 windiest days (m/s)**	**Max wind speed (m/s)**
Brandberg/Omaruru River/ Spitzkoppe/	Omaruru	150/96/ 89	3	NDJ	2.04	5.96	8.06
Tinkas River/Remhoogte/ Hauchabfontein	Gobabeb	97/129/ 146	23	JFM	3.37	7.51	9.20
Gorab	Mariental	168	12	DJF	1.01	5.18	7.66
Namtib	Diaz point	131	26	DJF	7.98	18.37	21.02
Carolinahof/Kliphoek/ Grunau	Keetmans-hoop	124/154/151	26	DJF	3.91	10.45	12.28

The quarter from which the average of the 5 windiest days and max wind speed values were derived was based on the months of the warmest quarter (i.e. PwarmQ). Values for the average of the 5 windiest days and max wind speed are for the windiest time of day (consistently 20 h00), with the exception of Diaz Point (14 h00).

Although there was considerable variability in geology and lithology within populations, leptosols predominated at most sites (Table 5). *Aloe dichotoma* populations were also strongly associated with rock outcrops in the north and with a range of other soil types such as regosols, calcisols, arenosols and cambisols, in more poleward populations.

**Table 5:** Geology (rock type) and lithology (soil type) associated with each population

**Population**	**Rock type**	**Soil type**
Brandberg	granite	leptosols/rock outcrops
Omaruru River	complex geology	leptosols/rock outcrops
Spitzkoppe	granite	leptosols/rock outcrops
Tinkas River	quartzite/granite	leptosols/rock outcrops
Remhoogte	quartzite/limestone	leptosols
Hauchabfontein	limestone	leptosols
Gorab	quartzite	leptosols/regosols
Namtib	granite	leptosols/regosols
Carolinahof	sandstones/limestones/shales	calcisols
Kliphoek	sandstone/shale	leptosols
Grunau	dolorite	leptosols/regosols
Bulletrap	gneiss	leptosols
Rooifontein	gneiss	arenosols/leptosols
Gannabos	shale	cambisols

Populations above and below the bold line fall in the summer and winter rainfall zones, respectively.

**Figure 8:**
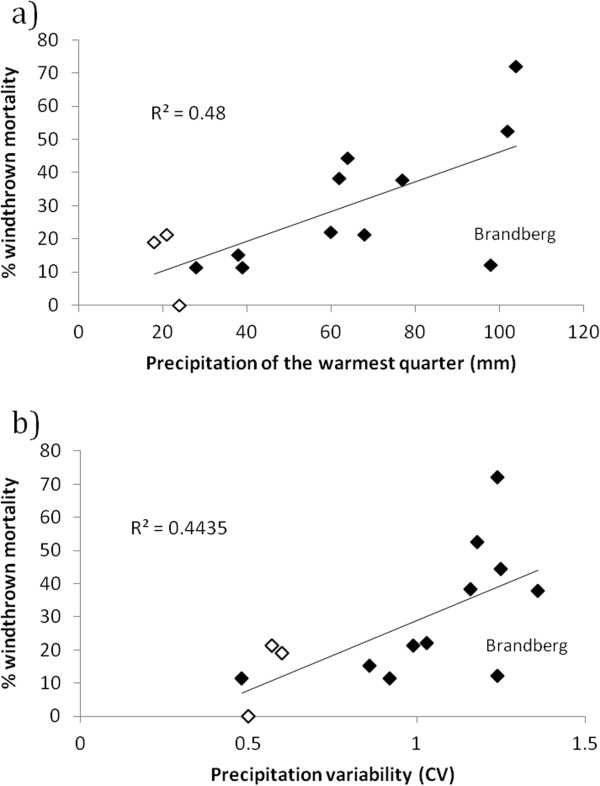
**Relationship between the percentage of windthrown individuals and (a) mean precipitation of the warmest quarter and (b) precipitation variability (co-efficient of variation), respectively. (a & b)** Black and white diamonds represent summer and winter rainfall zones, respectively, and the value for the high elevation Brandberg population is labelled in both figures. R^2^ values reported are for combined summer and winter rainfall zones.

Measures of wet and dry season rainfall were well correlated with the percentage of windthrown mortality (positively and negatively, respectively), while temperature measures were not. Specifically, mean precipitation of the warmest quarter (PwarmQ), which is a measure of summer precipitation, had a strong positive relationship with the percentage of windthrown mortality (R^2^ = 0.48, p = 0.01, n = 14; Figure 8a), as did variability in rainfall (R^2^ = 0.44, p = 0.02, n = 14; Figure 8b), which is generally greater within the SRZ. Since summer rainfall and rainfall variability in the WRZ are typically low compared to the SRZ (and would therefore influence the slope of the lines and strength of the correlations) relationships were assessed whilst excluding WRZ populations. This yielded R^2^ values of 0.45 for PwarmQ, and 0.37 for precipitation variability. Further exclusion of the anomalous high elevation Brandberg population from the SRZ improved R^2^ values to 0.81 and 0.54 for PwarmQ and precipitation variability, respectively.

### Logistic regression analysis

Notwithstanding shortcomings in the data, all available independent variables were combined into a logistic regression model to assess the relative influence of variables on windthrown mortality, as well as their combined predictive power (Table 6). Lithological data were chosen over geological data due to problems arising from co-correlation, as well as the assumed greater relevance of the former variable. Similarly, precipitation variability was excluded because it was highly correlated with PwarmQ, which had a stronger tie to the percentage of windthrown mortality.

**Table 6:** Logistic model results

**Independent variable**	**Odds ratio**	**Std. Err.**	**z**	**p**	**95% conf. interval**
Basal circumference	1.21	0.08	2.79	0.005	[1.06; 1.39]
PwarmQ	3.90	0.70	7.63	<0.001	[2.75; 5.53]
Ave. of the 5 windiest days	2.59	0.50	4.92	<0.001	[1.77; 3.79]
Pwarmq*Ave. of the 5 windiest days	3.85	0.90	5.73	<0.001	[2.43; 6.10]
Leptosols	4.86	1.54	4.99	<0.001	[2.61; 9.04]
Regosols	0.67	0.13	−2.06	0.039	[0.46; 0.98]
Constant	0.21	0.05	−6.39	<0.001	[0.13; 0.34]

The logistic model included continuous and categorical environmental variables, and illustrates the influence of main and interactive effects on windthrown mortality.

Results suggest that our model fitted reasonably, but not exceptionally well. A likelihood-ratio test against a null model was highly significant $\left(\chi_{4}^{2}=174.75,\;p<0.001\right),$ and the pseudo-R^2^ was 12.0%. Tests for goodness of fit and model specification both returned non-significant (i.e. favourable) results (Hosmer-Lemeshow goodness-of-fit test [34]: $\chi_{8}^{2}=7.25,p=0.51$; link test of squared predicted value *z* = − 0.51, p = 0.41). Using a cut-off of 0.25, the model correctly predicted 69% of observed windthrown dead (model sensitivity) and 67% of observed non-windthrown dead (model specificity). The resulting area under the ROC curve was 0.73 (compared to 0.5 obtained with a null model).

Odds ratios for main effects can be interpreted as the change in the odds of windthrown mortality associated with a one standard deviation increase in the independent variable. Therefore all variables with an odds ratio greater than one (all except regosols) were associated with increasing the odds of windthrow mortality by a factor of between 1.21 (basal circumference) and 4.86 (leptosols). Being located on regosols, on the other hand, reduced the odds of windthrow mortality by a factor of 0.67.

The significant interaction between PwarmQ and the average of the 5 windiest days meant that simultaneous increases in these variables made a tree particularly susceptible to windthrow. If either PwarmQ or the average of the 5 windiest days increased by one standard deviation while the other was held constant at its mean, the odds of windthrown mortality increased 3.90 and 2.59 times, respectively. However, if both variables were simultaneously increased by one standard deviation, the odds increased by a factor of 3.90*2.59*3.85 = 38.89.

## Discussion

Windthrow is a previously unappreciated and important source of mortality in *A. dichotoma* populations. This is especially true for populations within the northern half of the SRZ, where (with the exclusion of the high elevation Brandberg population) windthrow accounted for an average of just under half (49%, n=5) of observed mortality. Even if one accepts a certain degree of error due to possible misclassifications of windthrow (as addressed in the methods section), this still represents a considerable proportion of total mortality.

With the exception of the south-western corner of Namibia, which receives frontal rainfall in winter, the SRZ receives its rainfall predominantly through convective thunderstorms [20]. These storms originate in the north and east as a result of moisture fed into the system by the southerly position of the Intertropical Convergence Zone during the austral summer season [35]. Storm systems typically move southwards and westwards, accompanied by strong winds and heavy localised showers [36]. This combination of strong wind gusts and associated heavy rain are thought to promote localised windthrow events through the following mechanism. After initially strong gusts of wind that herald the arrival of the storm, heavy downpours saturate the soil and increase its malleability. This is followed by rapid absorption and storage of water in the spongy pith of the stem, canopy branches and leaves of *A. dichotoma* individuals, making trees (especially large adults) top-heavy after heavy precipitation events. The characteristically rigid canopy structure in *A. dichotoma*, coupled with shallow rooting depths and/or rooting in unstable soil horizons then make individuals prone to windthrown mortality during strong gusts of wind. Such gusts may be associated with the current or - more likely - subsequent storm event/s which often arrive before the soil has dried sufficiently to lose its malleability.

Reproductively important mature adults and senescent individuals appeared to be particularly susceptible to windthrown mortality, judging from the similarity in size between windthrown individuals and living mature adult or senescent trees, as well as the increased incidence of windthrown mortality in populations with proportionally more adults. Results from the logistic regression model, too, indicated an increase in the odds of windthrown mortality as basal circumference increased.

Notwithstanding the influence of size on windthrow susceptibility, there can be little doubt that *A. dichotoma’s* more or less rigid architectural structure is also a significant contributor toward the species’ predisposition to being windthrown. While most trees tend to mitigate against the drag forces generated by strong winds through flexing in their stems and branches (notwithstanding oscillations that may develop in synchrony with wind gusts) and re-orientating their leaves to reduce drag [27], this strategy for energy dissipation is not possible in *A. dichotoma* due to the relative inflexibility of its stem, branches and leaf rosettes. This exposes the species to much greater drag forces, especially with respect to larger mature adult and senescent individuals with denser canopies, compared to smaller unbranched juveniles.

Compounding this vulnerability is the typically large height difference between mature *A. dichotoma* individuals and the surrounding vegetation, as well as the species’ apparent inability to form closed canopy stands. These population level characteristics have been well studied in commercial forestry species where tapering vegetation heights at the stand-edge and thinning of in-stand canopies have been shown to reduce the chances of windthrow [1,37].

Given the strong indication that windthrown individuals are derived from the larger mature adult and senescent age classes [23,24] (Figure 5, this study), the poor relationship between the surface area of living mature adult individuals and windthrown mortality was surprising. This is at least partially attributable to the relatively small number of populations sampled, but the greater reason probably has to do with micro-topographical and -lithological variability within each population, which creates pockets sheltered from the wind, and/or areas conducive to more secure rooting. It is also conceivable that above an as yet undetermined surface area threshold the likelihood of being windthrown becomes more or less equal. In these instances, environmental factors (such as those mentioned above) again become the chief control in determining whether windthrow occurs, and not surface area.

The vulnerability of adult and senescent *A. dichotoma* individuals within certain populations to windthrown mortality is cause for concern, as these age classes, particularly the adults, hold a disproportionate amount of the reproductive potential in a population. Moreover, the characteristically infrequent nature of recruitment, estimated at intervals of 10 to 15 years or more in *A. dichotoma* populations growing in more marginal habitats [17,19] (M.T. Hoffman & S. Jack, unpublished observations), means that these populations are vulnerable to windthrow events which might occur on a more regular basis. Windthrow can therefore be an important influence on population structure, and ultimately, on the long term persistence of populations. Smaller, marginal populations are at an especially high risk of becoming locally extinct as field observations and anecdotal evidence suggest that it is not unusual for more than a dozen individuals to be blown over during a single storm event (e.g. pers. comm.: Mr. Robert de Pauw, Kenhardt, 26/02/2008).

Of the physical and climatic variables investigated as drivers of windthrow, mean precipitation of the warmest quarter (PwarmQ) (i.e. the amount of rain which falls in summer) and precipitation variability emerged as the two single variables best explaining spatial patterns in the percentage of windthrown mortality. In both cases, a partial reason for the strong relationship was due to the difference in summer rainfall amount and consistency received in equatorward summer and poleward winter rainfall zones, respectively. But even when WRZ populations were removed from the analysis the relationships were still good, and they became excellent when the anomalous high elevation Brandberg population was excluded. This suggests that the greater percentage of windthrown deaths in the Namibian SRZ is attributable, at least in part, to a more intense period of summer rainfall, with a likely associated higher frequency and lower return interval of high rainfall events.

The multivariate analysis, which focussed on the SRZ due to the higher incidence of windthrown mortality in this region, supported this basic conclusion, although with some interesting additional findings. When co-correlated variables were discarded, the main effects contributing to windthrown mortality came chiefly from leptosols, PwarmQ and the average of the 5 windiest days per month. However, while leptosols had the largest odds ratio, this variable had a comparatively poor z-score and a high standard error, advising a cautious interpretation. On the other hand, the higher z-scores and lower standard error values associated with PwarmQ and the average of the 5 windiest days per month suggested greater confidence in the odds ratios accompanying these variables. This indicated that summer rainfall, but also accompanying wind speeds, were responsible for catalysing windthrow events. Interestingly, despite the paucity in wind speed records, when the average of the 5 windiest days per month was combined with PwarmQ to create a new interaction term, this yielded a significant positive effect on windthrown mortality (the only significant interaction term). This result, along with the positive effect from leptosols and basal circumference, suggested that windthrow events were influenced to a large degree by the combined effect of summer rainfall intensity and associated high winds, as well as local lithology and tree size. While this conclusion matches our observations in the field, it is important to note that not all of the data are of a good spatial resolution, and that improvements in this regard would greatly aid our confidence in these conclusions.

This hypothesis for mortality in *A. dichotoma* is the antithesis of previous ideas, which link mortality to water balance constraints due to rising temperatures [17], or severe and prolonged drought events [19], and instead indicates that a high percentage of mortality is related to brief periods when there is a *surplus* of water. This study also provides some preliminary evidence that windthrown mortality might have a more recent temporal signature than ‘standing’ mortality, but there is little evidence to suggest an increase in more recent windthrown mortality associated with increases in extreme weather events e.g. [21,22]. Instead, the peak in windthrown mortality estimated at around 40 years ago is likely to be tied to a well documented period of extremely wet conditions (and associated storminess) during the mid-1970s, which saw widespread flooding in southern Africa [20]. Conversely, the peak in non-windthrown ‘standing’ mortality which occurred as much as sixty years ago, may be remnants of particularly severe drought conditions which prevailed during the early 1930s and 1940s [19,20]. While an interpretation of these temporal dynamics is necessarily speculative, an increase in the number of sampled populations, more temporally consistent sampling, refinements of the ‘time since death’ technique of aging dead skeletons (from, for example, additional repeat photographs), and a better understanding of the physiological thresholds which result in mortality, will help resolve temporal questions around mortality, as well as whether windthrown mortality is increasing or not.

## Conclusions

The present study has highlighted windthrow as an important form of mortality in *A. dichotoma*, especially given that it disproportionately affects reproductively mature adult individuals in a species known to recruit infrequently. In addition, the study has broadly defined the climatic and physical conditions which lead to a higher prevalence in windthrown mortality, and illustrated where the above conditions intersect to increase the likelihood of windthrow events. However, the available data resolution makes it difficult to make spatially precise predictions about which populations are likely to be most at risk. Moreover, the modelling exercise demonstrates that threshold conditions required for the occurrence of localised windthrow events necessarily involve the combined influence of several variables operating in concert, and threshold conditions are likely to be dynamic. For example, a decrease in one or more variables can be compensated for by an increase in others with the same net result in terms of probability of windthrown mortality. Therefore, despite increases in the spatial resolution of data, it may be difficult to determine where and how threshold conditions will align to catalyse windthrow.

Despite these caveats, we can be reasonably sure that populations in the equatorward SRZ which receive higher amounts and/or frequency of convective rainfall and the necessary wind and lithological conditions appear to be at a greater risk of windthrow. Populations occurring within this latitudinal band may, therefore, benefit from more frequent and active monitoring efforts. Smaller, geographically isolated and more climatically marginal populations dominated by adults should be of the highest monitoring priority, as the long term persistence of these populations is most seriously threatened.

## Competing interests

The authors declare that they have no competing interests.

## Authors’ contributions

Field observations and discussion between SLJ, MTH and RFR prompted the need for an investigation of windthrown mortality. SLJ performed the fieldwork. Data were analysed by SLJ, ID and MA. SLJ wrote the manuscript, with input and comment from all authors. All authors read and approved the final manuscript.
